# Development and validation of the self-management profile for type 2 diabetes (SMP-T2D)

**DOI:** 10.1186/1477-7525-10-125

**Published:** 2012-10-05

**Authors:** Mark Peyrot, Donald M Bushnell, Jennie H Best, Mona L Martin, Ann Cameron, Donald L Patrick

**Affiliations:** 1 Department of Sociology, Loyola University Maryland, Baltimore, MD, USA; 2 Health Research Associates, Inc, Mountlake Terrace, WA, USA; 3 Amylin Pharmaceuticals, Inc, San Diego, CA, USA; 4 Seattle Quality of Life Group, Department of Health Services, University of Washington, Seattle, WA, USA

## Abstract

**Background:**

This study evaluated the measurement properties of a newly developed instrument – the Self-Management Profile for Type 2 Diabetes (SMP-T2D).

**Methods:**

The 18-item SMP-T2D assesses 12 constructs: level and perceived ease of performance in five self-care domains (blood glucose monitoring, medication-taking, healthy eating, being physically active, and coping), and two global constructs (ease of weight management, confidence with ability to manage diabetes). Validation analyses were based on two studies involving 240 patients with T2D, Study 1 (Clinicaltrials.gov #NCT00637273) with SMP-T2D administration supplemented by SMP-T2D retest one week later, and Study 2 (Clinical trials.gov #NCT00877890) with SMP-T2D administration supplemented by 24-week SMP-T2D follow-up after medication change. Validation included clinical indicators and measures of patient reported quality of life, psychological well-being and treatment outcomes.

**Results:**

All multi-item SMP-T2D measures showed acceptable internal consistency (alphas = 0.71 to 0.87); ten measures had test-retest reliability >0.75. Correlations among SMP-T2D measures and between SMP-T2D measures and validation measures, which were as hypothesized, provided evidence of convergent and discriminant validity. Scores for six SMP-T2D measures improved significantly during Study 2. Multiple regression analysis showed independent associations between change in SMP-T2D measures and change in trial outcomes from baseline to end-of-study.

**Conclusions:**

Two studies provide preliminary evidence regarding the reliability, validity and responsiveness of the SMP-T2D. Further research on the utility of the instrument is needed.

## Background

Type 2 diabetes mellitus can have substantial effects on patients’ physical and psychosocial well-being. People with diabetes, their families and health care providers, and health policymakers are interested in improving diabetes outcomes, which requires both medical care and patient self-management. As such, the American Diabetes Association (ADA) Standards of Medical Care has identified self-management education and ongoing support as integral components of diabetes care, and recommends monitoring self-management behaviors in patients receiving treatment for diabetes [1].

Given the importance of patient self-management in the control of diabetes, evaluations of effectiveness in clinical trials of behavioral and pharmacologic treatments should include an assessment of diabetes self-management, augmenting physiological outcomes such as blood glucose and patient reported outcomes (PROs) such as health-related quality of life. Better self-management is expected to lead to improved glycemic control and better patient outcomes in weight management and diabetes-related distress. Therefore, we initiated a project to develop and validate a new patient self-report instrument, the Self-Management Profile for Type 2 Diabetes (SMP-T2D), which would assess multiple domains and dimensions of self-management and would be brief enough for use in clinical trials.

The US Food and Drug Administration (FDA) has provided guidance on the development of PRO instruments for use in evaluating medical products for labeling claims [2]. Data used for labeling claims must meet the most stringent standards and these standards define best practice. The FDA guidance requires documentation of patient input into instrument development, and the development of the SMP-T2D used patient input to assess the relevance of the concepts reflected in the instrument as well as patient comprehension and interpretation of individual items.

This paper reports the development of the SMP-T2D and the results of two studies evaluating the measurement properties of the SMP-T2D in the clinical trial setting.

## Methods

### Concept development

Concepts to be included in the SMP-T2D development process were based on a systematic review of the diabetes self-management literature. The American Diabetes Association and the American Association of Diabetes Educators (AADE) have developed standards for the provision and evaluation of diabetes self-management education and support [1,3]. These standards identified seven domains (the AADE7) that are the essential components of self-management: physical activity, healthy eating, medication taking, blood glucose monitoring, problem solving, risk reduction, and coping (psychosocial adaptation) [4,5]. A self-administered questionnaire, the Diabetes Self-Management Assessment and Report Tool (D-SMART), was developed to assess the AADE7 [6-8].

The D-SMART was developed to provide a detailed assessment that could be used to guide comprehensive self-management education. But this instrument is too lengthy to be used in clinical trials of diabetes treatment medications. Of the seven AADE domains, two have less relevance to the clinical trial situation. Risk reduction involves efforts to prevent long-term complications, e.g., by having periodic tests and examinations, and actions such as vaccinations, prophylactic aspirin, foot checking, etc. Although important, these are not targets of diabetes medication trials. Problem solving relates to dealing with situations that are likely to be covered by clinical trial protocols and/or to be managed by clinicians on the research team. Therefore, these two domains were not included in the instrument development process.

The D-SMART also incorporates dimensions believed to be important in patient implementation of self-management, including confidence in managing diabetes, barriers to self-care, and self-care goals. While patient goals are important in routine clinical care, in clinical trials the goals are generally defined by the trial, and so the SMP-T2D was not designed to assess personal goals. The concept of barriers has been identified as an important factor in self-management [6-10]. Instead of an inventory of barriers that includes multiple questions (as in the D-SMART), we focused on the *consequences* of barriers for *patients*, i.e., the degree to which barriers make it difficult to implement self-management. Self-management difficulty has been used as a way of measuring self-management performance [11]; this strategy recognizes that patients may implement self-management even though it is difficult, but the behavior is less likely to be sustained when it is difficult. Moreover, patients may be more willing to admit to having difficulty with self-management than to admit not implementing their prescribed regimen [12].

Self-efficacy has been viewed as a key construct in human behavior and was identified as another essential component in our measure of self-management [13,14]. Finally, in an effort to develop personalized weighting for the SMP-T2D measures, we included importance ratings for the five behavioral domains [15].

Following the identification of concepts from the existing literature, we developed a focus group interview guide to assess the relevance of the identified concepts and to identify new concepts. Four focus groups were conducted with a total of twenty-four participants with type 2 diabetes. Participants had a diagnosis of diabetes ranging from 1.5 to 30 years, with an average of 11.2 years. Most participants (66.7%) had an A1c between 7 and 11. Participants were taking either injectable (n = 12) or oral (n = 12) diabetes medications. The specific medication regimens varied greatly among participants.

One of the authors (MM) developed an initial coding framework from the focus group transcripts that linked to the literature and existing knowledge about self management. Next, two interviewers were trained to code concepts using this initial coding framework. After the first two transcripts were coded, ATLAS.ti software [16] was used to tag the assigned concept codes in each transcript. Results were reviewed by the coding team, and revisions to the coding framework were made keeping the focus group data in mind. Saturation was reached after the third focus group, as no new concepts emerged.

### Item generation and cognitive interviews

Construction of the SMP-T2D instructions and items followed recommendations regarding cognitive burden, response format and layout, and question order [17]. The initial draft of the SMP-T2D was mapped to patient expressions and quotations from the qualitative focus group data. Nine items measured the five key AADE7 domains: frequency of Monitoring Glucose non-adherence (1 item), frequency of Medication Taking adherence (1 item), frequency of Eating Healthy (2 items), frequency of Physical Activity (3 items), and consequences of Coping (2 items). Seven items measured the *difficulty* of performing the behaviors representing the five key AADE7 domains: Glucose Monitoring (1 item), Medication Taking (1 item), Eating Healthy (3 items), Physical Activity (1 item), and Coping (1 item). Two items measured more global constructs: difficulty with Managing Weight (1 item) and Confidence with Ability to Manage Diabetes (1 item).

The behavior measure for the concept of coping uses the same measurement logic as the D-SMART measure of coping [6], assessing the *consequences* of coping (i.e., amount of diabetes-related frustration and worry about future health) rather than coping behavior per se. Assessing *coping behavior* would require an inventory of effective coping strategies that would be beyond the scope of a measure to be used in clinical trials. The Ease of Coping item specifically refers to “coping with frustration and worry related to your diabetes.”

The “Ease of Managing Weight” was an outcome or benefit not contained in the AADE7. The content supporting this item emerged during the qualitative phase of the project as highly relevant to patients. Since this concept is also identified in the ADA guidelines as an important aspect of diabetes self-management, it was included in the instrument.

During the development phase, an importance item (“How important is it for you right now to…”) was included for each of the five behaviors and ease of managing weight in order to assess the potential of deriving weights for each domain score.

Cognitive interviews [18] were conducted with 34 patients with type 2 diabetes to assess the comprehension and interpretation of the draft instrument. The draft used in the validation studies incorporated revisions based on that process (see Appendix). Participants in the cognitive interviews had a diagnosis of diabetes ranging from 1 to 30 years, with an average of 10.7 years. Most participants (76.5%) had an A1c between 7 and 11. The specific medication regimens varied greatly among participants; 17 participants were using oral medications only, 16 participants were using injectable medications alone or in combination with oral medications, and one participant was managing diabetes with diet and exercise.

### Scoring

All SMP-T2D scores were transformed to a 0–100 scale, with equal increments between responses. Scoring was designed so that higher scores indicate better self-management (as a result, items rating “difficulty of…” are reverse scored and labeled as “ease of…”). Except for the Physical Activity behavior measure scores for domains that had more than a single item were calculated as the mean of the available items. The measure of Physical Activity behavior consists of 3 items, one each addressing light, moderate, and vigorous activity. Scoring followed the logic of the Rapid Assessment of Physical Activity (RAPA) [19], where respondents’ scores are categorized into one of four levels of physical activity: sedentary (0 = no days of vigorous or moderate activity, and less than 2 days of light activity); underactive, light activity (33.3 = no days of vigorous activity with 1–2 days of moderate activity and/or 2 or more days of light activity); underactive, regular activity (66.7 = 1–2 days of vigorous activity and/or 3–4 days of moderate activity); and active (100 = 3 or more days of vigorous activity and/or 5 or more days of moderate activity).

### Participants and procedures for validation studies

There were two validation studies; participant characteristics are reported in Table 1. In Study 1 the SMP-T2D was administered at the end of an ongoing clinical trial to evaluate its measurement properties. The parent trial was a randomized three-arm study designed to compare the effects of exenatide once weekly (QW) to sitagliptin and pioglitazone over 26-weeks [20,21]. Glucose monitoring was not specified by protocol.

**Table 1:** Sample characteristics

**MEASURE**	**N (%) or Mean (SD)**
**STUDY 1**	147 (100%)
Gender	
Male	88 (59.9%)
Female	59 (40.1%)
Ethnicity	
Caucasian	88 (59.9%)
Black	24 (16.3%)
Hispanic	21 (14.3%)
Asian	11 (7.5%)
Other	3 (2.0%)
Age (at enrollment)	54.6 (10.8)
Treatment Assignment	
Sitagliptan	64 (43.5%)
Pioglitazone	44 (29.9%)
Exenatide QW	39 (26.5%)
BMI (kg/m^2)	33.7 (5.3)
A1c	8.5 (1.2)
DTSQs frequency of perceived hyperglycemia (0–6)	2.8 (1.7)
DTSQs frequency of perceived hypoglycemia (0–6)	0.9 (1.5)
WHO-5 Well-Being Index total (0–100)	67.6 (18.1)
EQ-5D Visual Analog Scale (0–100)	76.2 (18.4)
EQ-5D Item 3 – Usual Activities (1–3, higher score = more problems)	1.2 (0.4)
IWQOL-Lite total score (0–100)	82.6 (15.5)
**STUDY 2**	93 (100%)
Gender	
Male	61 (65.6%)
Female	32 (34.4%)
Ethnicity	
Caucasian	76 (81.7%)
Black	12 (12.9%)
Other	5 (5.4%)
Age (at enrollment)	50.8 (10.5)
Medication (at enrollment)	
Metformin only	61 (65.6%)
Metformin and TZD	10 (10.8%)
TZD only	1 (1.0%)
None	21 (22.6)
Treatment assignment (Exenatide dose)	
2 mg, once weekly	22 (23.7%)
5 mg, once monthly	22 (23.7%)
8 mg, once monthly	26 (28.0%)
11 mg, once monthly	23 (24.7%)
Baseline BMI (kg/m^2)	34.1 (5.7)
Baseline A1c	8.3 (1.1)
Baseline DTSQs perceived hyperglycemia	2.8 (1.9)
Baseline DTSQs perceived hypoglycemia	1.0 (1.3)
Change from baseline in BMI (kg/m^2)	−0.4 (5.3)***
Change from baseline in A1c	−1.2 (1.2)***
Change from baseline in DTSQs perceived hyperglycemia	−1.1 (2.1)***
Change from baseline in DTSQs perceived hypoglycemia	0.2 (1.6)

*BMI*, body mass index; *A1c*, glycated hemoglobin; *SD*, standard deviation; *** p <; 0.001 (two-tailed).

Participants were adults diagnosed with type 2 diabetes and treated with metformin. A1c values at screening could range between 6.5% and 10.5% with body mass index (BMI) ranging between 25 and 45 kg/m^2^. Participants previously treated with other diabetes medications were excluded.

The Study 1 protocol was amended to include administration of the SMP-T2D after some participants had already completed the 26-week controlled study; 147 of the 243 English-speaking participants who had not completed the trial at 26 weeks were administered the SMP-T2D at week 26 (for cross-sectional validation) and week 27 (for reproducibility assessment). These 147 participants comprise the study population for this study.

For Study 2 the parent trial was a randomized four-arm study designed to compare the effects of different doses of exenatide once weekly or once monthly [22]. Glucose monitoring was not specified by protocol. Participants were 93 adults diagnosed with type 2 diabetes, who either were not taking diabetes medication or were treated with metformin and/or thiazolidinedione. A1c values at screening could range between 7.1% and 11.0%, and body weight was stable for at least 3 months prior to screening.

### Validation measures

All measures of constructs used to evaluate the construct validity of the SMP-T2D were those included in the parent study protocols and were obtained at the same times the SMP-T2D was administered (except for the SMP-T2D retest in Study 1). These trials did not have parallel measures of the constructs assessed by the SMP-T2D (e.g., adherence, self-efficacy, barriers, etc.).

Clinical assessments in both studies included clinician-reported BMI and the biomarker A1c. In Study 1 five PRO construct validity instruments were administered. *EuroQoL-Five Dimensions (EQ-5D)*[23]: the validation analyses used only the item measuring severity of problems performing usual activities (which was treated as a set of ordered categories for analysis purposes) and the visual analog scale (VAS) measuring patients’ perceived health status, the former because it provides validation for physical activity domain and the latter because the VAS has greater sensitivity than the health utility index [24]. *Impact of Weight on Quality of Life Questionnaire, Lite Version (IWQOL-Lite)*[25]: the validation analyses used only the total score, ranging from 0 (low) to 100 (high). *World Health Organization-Five Well-being Index (WHO-5)*[26]: scores were standardized to a 0–100 range, with higher scores representing positive psychological well-being. *Diabetes Treatment Satisfaction Questionnaire (Status Version, DTSQs)*[27]: the validation analyses used only the items measuring perceived frequency of hypoglycemia and perceived frequency of hyperglycemia. These DTSQs items were also used in Study 2.

All studies were approved by an Institutional Review Board and were conducted in accordance with ethical principles described in the Declaration of Helsinki.

### Statistical analyses

All analyses were performed using the Statistical Package for the Social Sciences (SPSS), Release 11.5.0 [28].

#### Descriptive statistics

In addition to the mean and standard deviation for each SMP-T2D measure, the percentage of persons giving the minimum and maximum scores were used to determine floor and ceiling effects.

#### Internal consistency reliability

For the measures that contained multiple items, Cronbach’s alpha [29] was used to assess the degree to which the set of items measured a single unidimensional latent construct. An alpha equal to or greater than 0.70 is defined as adequate internal consistency [30].

#### Test-retest reliability

The extent to which the SMP-T2D measures have stable scores over time in the absence of true change was assessed with two administrations of the SMP-T2D at a one-week interval where change was not expected. One test used the average Spearman-Brown coefficient; a coefficient equal to or greater than 0.70 is defined as adequate test-retest reliability [30]. The t-test was used for the change in mean scores over time (represented in baseline standard deviation units to permit an assessment of effect size [31]); smaller change indicates greater reliability, and a difference of >0.5 standard deviation units indicates a minimum detectable difference [32].

#### Construct validity

Construct validity was assessed through correlations between measures representing constructs that were hypothesized to be more strongly (convergent) or more weakly (discriminant) related according to *a priori* expectations based on the theoretical relationships among constructs [33].

The first phase of validity analysis examined a correlation matrix of associations among the SMP-T2D measures; this analysis was performed only for the Study 1 data because the Study 2 data was regarded as redundant*. Measures for each of the five behaviors were predicted to be more strongly associated with their corresponding measures of perceived ease than with other items that did not correspond with each other*. Two more specific convergent/discriminant validity hypotheses were formulated: *(1) Ease of Managing Weight will be more strongly associated with Ease of Eating Healthy and Ease of Physical Activity than with Ease of Medication Taking Adherence and Ease of Glucose Monitoring, and the association with Ease of Coping would be intermediate; (2) Confidence with Ability to Manage Diabetes will be more strongly associated with Coping and Ease of Coping than with measures of behavior or perceived ease from other domains.*

The second phase of validity analysis examined the associations between the SMP-T2D measures and the construct validity measures*. All SMP-T2D measures were predicted to be positively associated with better scores on all construct validity measures*. However, *construct validity measures were predicted to be more strongly associated with lifestyle domains (Eating Healthy, Physical Activity and Coping) than with medical domains (Medication Taking and Glucose Monitoring);* rationale – in a clinical trial setting, medication taking is constrained and the other factors are likely to have greater impact on outcomes, or to be affected by those outcomes. Moreover, *construct validity measures were predicted to be more strongly associated with measures of perceived ease of behavior than measures of behavior frequency*; rationale – ease of a behavior is a more sensitive indicator of the degree to which a behavior is performed and may be less subject to reporting bias due to social desirability (i.e., it is easier to admit to difficulty than non-adherence). Finally, *construct validity measures were predicted to be more strongly associated with Coping measures, Confidence in Ability to Manage Diabetes and Ease of Managing Weight than with frequency and perceived ease of Medication Taking, Glucose Monitoring, Eating Healthy and Physical Activity*; rationale – the former measures represent more global constructs that incorporate multiple specific behavioral domains.

#### Ability to detect change

Ability of the instrument to detect change over time was assessed in Study 2. All Study 2 participants were changing their medication and were included in the analyses. The first type of analysis assessed change in mean SMP-T2D scores over time. *It was hypothesized that there would be improvement in the Eating Healthy and Coping measures, Ease of Managing Weight and Confidence in Ability to Manage Diabetes but not other SMP-T2D measures*; rationale – the effects of exenatide are strongest for eating, weight and glycemic control [34] (the intervention did not target glucose monitoring or physical activity, and patients switched from oral to injectable medication).

The second type of validity analysis assessed the association between change in SMP-T2D scores and change in validation measures over the course of the study. *Validity measures were predicted to be most strongly associated with the Coping measures, Confidence in Ability to Manage Diabetes and Ease of Managing Weight than with other measures*; rationale – the former measures represent more global constructs that incorporate multiple specific behavioral domains. *BMI was predicted to be more strongly associated with dietary and physical activity measures than with measures of glucose monitoring and medication taking*; rationale – weight is more dependent on caloric intake and expenditure than other self-management behaviors.

A supplementary assessment of ability to detect change used a stepwise regression analysis of the change scores for each of the 4 validity measures to determine the independent relationships with SMP-T2D change scores, controlling for the baseline value of the validity measure.

## Results

The time needed to complete the SMP-T2D across patients ranged from 3 to 5 minutes. Readability analyses of the pre-final SMP-T2D were conducted in Microsoft Word® 2003 and showed a Flesch-Kincaid Grade Level score of 7.0.

Preliminary analysis of the six importance ratings in Study 1 showed all domains being equally “Very Important” and therefore these items were not examined further. These items were not included in Study 2.

### Scaling

Means for SMP-T2D measures ranged between 62 and 97 across the two studies; only the Medication Taking frequency and ease measures had means above 85 (see Table 2). Floor effects were low, with a high of 11.8% having the minimum score for Glucose Monitoring behavior in Study 2 (interpolated median for both studies = 2.5%). Ceiling effects were more pronounced, with a high of 89.2% having the maximum score for Ease of Medication Taking in Study 2 (interpolated median for non-medication domains in both studies = 33.0%).

**Table 2:** SMP-T2D measurement model

**Concept (Questionnaire Item No.)**	**Study 1**				**Study 2**		
	**Baseline** **Mean (SD)**	**% Min Score,% Max Score**	**Test-Retest Reliability**†	**Test-Retest Shift**‡	**Baseline** **Mean (SD)**	**% Min Score,% Max Score**	**During-Study Change**‡
**Patient behavior domains:**							
Monitor blood glucose (#3)	81.24 (24.68)	2.0, 49.7	0.86	0.25*	72.19 (34.12)	11.8, 44.1	0.11
Take medications as prescribed (#2)	96.70 (8.86)	0.0, 84.4	0.41	0.00	90.01 (21.25)	2.2, 71.0	-.09
Eat healthy (#4, #5)	65.16 (24.00)	3.4, 3.4	0.91	0.02	64.21 (25.35)	2.2, 5.4	0.35***
Engage in physical activity (#6, #7, #8)	69.16 (30.24)	4.8, 40.1	0.63	0.05	63.44 (30.72)	5.4, 32.3	−0.02
Cope with frustration and worry (#10, #11)	76.66 (22.07)	1.4, 25.2	0.80	0.15	72.04 (21.52)	0.0, 15.1	0.59***
**Ease of patient behaviors:**							
Monitoring blood glucose (#9a)	84.93 (26.01)	4.1, 66.4	0.81	0.04	87.50 (26.34)	5.4, 75.0	0.02
Taking medications as prescribed (#9b)	92.81 (19.69)	2.7, 82.9	0.78	0.17	95.16 (17.00)	2.2, 89.2	0.05
Eating healthy (#9d, #9e, #9f)	72.76 (23.11)	1.4, 18.4	0.86	0.06	71.42 (23.45)	1.1, 16.1	0.20*
Engaging in physical activity (#9 g)	69.22 (30.28)	5.4, 36.7	0.87	0.06	72.04 (28.52)	4.3, 37.6	0.24*
Coping with frustration and worry (#9 h)	78.57 (27.13)	4.8, 48.3	0.79	0.03	80.11 (24.61)	2.2, 50.5	0.16
**Ease of managing weight (#9c)**	64.12 (31.52)	8.2, 29.3	0.88	0.04	68.82 (34.11)	8.6, 43.0	0.35***
**Confidence in ability to manage diabetes (#12)**	66.44 (26.00)	4.1, 22.6	0.88	0.09	62.90 (25.17)	2.2, 16.1	0.47***

† Test-retest reliability measured by Spearman-Brown coefficient, 1-week reproducibility.

‡ Change in mean from baseline represented in baseline standard deviation units: * p <; 0.05 (two-tailed); ** p <; 0.01 (two-tailed); *** p <; 0.001 (two-tailed).

### Internal consistency

The SMP-T2D profile contains four multi-item measures. Inter-item agreement for these measures was assessed by alpha in Study 1 and Study 2. Alphas ranged from 0.71 to 0.87 (median = 0.80). (see Table 2).

### Test-retest reliability

In Study 1, ten SMP-T2D measures had Spearman-Brown coefficients greater than 0.77 (see Table 2); reproducibility at 1 week ranged from 0.41 for Medication Taking behavior to 0.91 for Eating Healthy behavior (interpolated median = 0.83). Only one measure (Glucose Monitoring behavior) changed significantly across administrations; the effect size was 0.25 which is half the size of the minimum detectable difference [32].

### Intercorrelations of SMP-T2D measures

Table 3 presents the associations within and between the domains of the SMP-T2D in Study 1. All convergent/discriminant validity hypotheses internal to the instrument were confirmed. As expected, the corresponding ease and behavior measures had stronger associations than those that do not correspond; all corresponding measures but only 2 of 20 non-corresponding measures correlated at p <; 0.001. Secondly, Ease of Managing Weight was more strongly associated with Ease of Eating Healthy and Ease of Physical Activity than with measures of perceived ease from other domains. Finally, Confidence with Ability to Manage Diabetes was more strongly associated with consequences of Coping and Ease of Coping than with measures of behavior or perceived ease from other domains.

**Table 3:** Correlations among SMP-T2D Measures in Study 1

**Patient behaviors to:**	**Ease of:**					**Ease of managing** **weight**	**Confidence in ability to manage diabetes**
	**Monitoring blood glucose**	**Taking medications as prescribed**	**Eating healthy**	**Engaging in physical activity**	**Coping with frustration and worry**		
Monitor blood glucose	0.40***	0.28**	0.06	0.03	0.09	0.09	0.09
Take medications as prescribed	0.24**	0.29***	0.13	0.14	0.18*	0.19*	0.21*
Eat healthy	0.15	0.05	0.35***	0.28**	0.20*	0.28**	0.28*
Engage in physical activity	−0.02	0.08	0.05	0.40***	0.11	0.03	0.10
Cope with frustration and worry	0.26**	0.13	0.36***	0.34***	0.69***	0.34***	0.63***
Ease of Managing Weight	0.18*	0.20*	0.57***	0.45***	0.40***	1.00	0.36***
Confidence in ability to manage diabetes	0.27**	0.21*	0.35***	0.38***	0.63***	0.36***	1.00

* Correlation is significant at the 0.05 level (2-tailed).

** Correlation is significant at the 0.01 level (2-tailed).

*** Correlation is significant at the 0.001 level (2-tailed).

### Convergent and discriminant validity

Table 4 presents the associations between baseline scores on the SMP-T2D measures and the construct validity measures in Study 1. All convergent/discriminant validity hypotheses were generally confirmed, although there were a few exceptions to each of the predicted patterns. Nine of twelve SMP-T2D measures were significantly associated with one or more validity measures and all significant associations were in the predicted direction.

**Table 4:** Validity correlation matrix from Study 1

	**A1c**	**DTSQs Perceived Frequency of Hyperglycemia**	**DTSQs Perceived Frequency of Hypoglycemia**	**Body Mass Index**	**IWQOL** **Total Score**	**EQ-5D** **Usual Activities** **(Item 3)**^**†**^	**EQ-5D Visual Analog Scale**	**WHO-5** **Total Score**
**Patient behaviors to:**								
Monitor blood glucose	0.03	0.04	0.13	−0.05	−0.05	−0.04	0.05	0.08
Take medications as prescribed	0.07	0.12	−0.05	−0.14	0.02	−0.07	0.06	0.16
Eat healthy	−0.02	−0.16	−0.04	−0.21*	0.21*	0.08	0.07	0.24**
Engage in physical activity	−0.03	0.03	0.03	−0.12	0.18*	−0.18*	0.05	0.28**
Cope with frustration and worry	−0.43***	−0.44***	0.11	−0.13	0.44***	−0.14	0.33***	0.45***
**Ease of:**								
Monitoring blood glucose	0.02	−0.10	0.07	−0.05	0.08	−0.17*	0.12	0.16
Taking medications as prescribed	0.07	0.11	0.05	−0.05	0.00	−0.14	0.08	0.16
Eating healthy	−0.09	−0.23**	−0.06	−0.09	0.20*	−0.03	0.10	0.31***
Engaging in physical activity	−0.17*	−0.23**	−0.10	−0.09	0.32***	−0.22**	0.26**	0.46***
Coping with frustration and worry	−0.26**	−0.29**	−0.06	−0.11	0.32***	−0.12	0.25**	0.46***
**Ease of managing weight**	−0.12	−0.18*	0.05	−0.33***	0.38***	−0.10	0.34***	0.37***
**Confidence in ability to manage diabetes**	−0.39***	−0.35***	−0.03	−0.10	0.34***	−0.04	0.28**	0.46***

Note: A1c, glycated hemoglobin; WHO-5, World Health Organization-Five Well-Being Index; EQ-5D, EuroQoL-Five Dimensions; DTSQs, Diabetes Treatment Satisfaction Questionnaire-Status Version; IWQOL, Impact Of Weight On Quality Of Life-Lite.

**†** EQ-5D item 3 (Usual Activities) used here is scored so that a higher score represents more severe problems.

* Correlation is significant at the 0.05 level (2-tailed).

** Correlation is significant at the 0.01 level (2-tailed).

*** Correlation is significant at the 0.001 level (2-tailed).

As predicted, all construct validity measures were more strongly associated with measures from the lifestyle domains (frequency and perceived ease of Eating Healthy, Physical Activity and Coping) than with measures from the medical regimen domains (frequency and perceived ease of Medication Taking and Glucose Monitoring). And most construct validity measures were more strongly associated with frequency and Ease of Coping than with frequency and perceived ease of Eating Healthy and Physical Activity. Additionally, with few exceptions all validity measures were more strongly associated with perceived ease of behavior than measures of behavior frequency. Finally, most construct validity measures were more strongly associated with Confidence in Ability to Manage Diabetes and Ease of Managing Weight than with frequency and perceived ease of Medication Taking, Glucose Monitoring, Eating Healthy and Physical Activity.

Eight SMP-T2D measures were significantly associated with one or more validity measures and all significant associations were in the predicted direction (see Table 5). For the four validity measures in common between Study 1 and 2, there was an 81% between-study concordance for statistical significance/non-significance of correlations with the SMP-T2D measures.

**Table 5:** Validity correlation matrix from Study 2

**SMP-T2D Measure**	**Baseline Scores**				**Change Scores** †			
	**A1c**	**DTSQs Perceived Frequency of Hyperglycemia**	**DTSQs Perceived Frequency of Hypoglycemia**	**Body Mass Index**	**A1c**	**DTSQs Perceived Frequency of Hyperglycemia**	**DTSQs Perceived Frequency of Hypoglycemia**	**Body Mass Index**
**Patient behaviors to:**								
Monitor blood glucose	.05	-.13	.04	.00	-.05	-.07	-.28**	-.14
Take medications as prescribed	-.16	-.07	.11	-.03	.11	.08	.02	.01
Eat healthy	-.21*	-.17	.08	-.17	-.24*	-.19	.02	-.22*
Engage in physical activity	.21*	.01	.02	-.02	-.10	.15	.11	-.03
Cope with frustration and worry	-.05	-.32**	.07	-.10	-.22*	-.15	.09	-.12
**Ease of:**								
Monitoring blood glucose	-.08	-.24*	-.11	-.02	-.04	-.08	-.03	-.03
Taking medications as prescribed	-.08	-.20	.01	-.03	.08	.00	.14	.05
Eating healthy	-.00	-.24*	.02	-.24	-.07	-.11	.04	-.02
Engaging in physical activity	.06	-.05	.15	-.23	-.02	-.18	-.08	-.23*
Coping with frustration and worry	-.03	-.23*	-.08	-.05	-.02	.04	.09	-.02
**Ease of managing weight**	.05	-.00	.08	-.47***	-.13	-.17	-.04	-.22*
**Confidence in ability to manage diabetes**	-.22*	-.23*	.20	-.07	-.31**	-.22*	-.00	-.16

Note: A1c, glycated hemoglobin; DTSQs, Diabetes Treatment Satisfaction Questionnaire-Status Version.

† Partial correlations controlling for baseline level of validity measure.

* Correlation is significant at the 0.05 level (2-tailed).

** Correlation is significant at the 0.01 level (2-tailed).

*** Correlation is significant at the 0.001 level (2-tailed).

### Ability to detect change

In Study 2 six of twelve SMP-T2D measures showed during-study improvement (see Table 2). As hypothesized, SMP-T2D measures relating to eating and weight, coping and confidence in managing diabetes exhibited statistically significant improvements (the only exception was Ease of Coping). Two measures (consequences of Coping behavior and Confidence in Managing Diabetes) had effect sizes near or above the 0.50 cut-off for a minimal detectable difference [32].

In Study 2, improvements in six SMP-T2D measures were associated with improvement in a trial outcome; none were associated with deterioration in an outcome (see Table 5).

Stepwise regression analysis of the change scores for each of the 4 validity measures assessed the independent relationships with change scores for the SMP-T2D measures, controlling for the baseline value of the validity measure. Change in Confidence in Managing Diabetes (beta = −0.27, p <; 0.01) and Eating Healthy behavior (beta = −0.20, p <; 0.05) accounted for 11.5% of the variance in change in A1c. Change in Physical Activity behavior and Glucose Monitoring behavior accounted for 11.2% of the variance in change of perceived frequency of hypoglycemia; these factors had offsetting effects, with increased physical activity associated with increased hypoglycemia (beta = 0.23, p <; 0.05) and increased Glucose Monitoring associated with reduced hypoglycemia (beta = −0.29, p <; 0.01). Change in Confidence in Managing Diabetes (beta = −0.16, p <; 0.05) accounted for 5.2% of the variance in change of perceived frequency of hyperglycemia. Change in Ease of Physical Activity (beta = −0.23, p <; 0.05) accounted for 2.5% of the variance in change in BMI.

## Discussion

Development of the SMP-T2D incorporated self-management concepts that patients with type 2 diabetes expressed as important in their experience with diabetes and its treatment. The specific items included in the SMP-T2D were generated using language as close as possible to that used by patients. Patients indicated a high level of understanding of the questionnaire items in cognitive interviews before the psychometric properties were assessed. This developmental process provides support for the content validity of the SMP-T2D and justification for the conceptual framework.

The results of the validation studies provide preliminary support for the reliability and validity of the SMP-T2D. Internal consistency for multiple-item measures was acceptable and test-retest reliability was adequate for all but two measures. The unexpected low test-retest reliability for the frequency of medication taking measure (days missed of prescribed diabetes medications during the past week) could be explained by the lack variability due to the clinical trial treatment context. The majority of participants adhered to their medication regimen, allowing outliers to skew the reliability estimate. This item should be retained for further research within later phase studies in which patient medication taking behavior is not as tightly controlled.

The modest test-retest reliability of the measure of physical activity behavior is surprising as the measure of time-specific reliability (inter-item agreement) was good. This may be due to the fact that the composite measure uses thresholds rather than continuous increments of the component items so that small changes in activity could yield substantial changes in scores. It is likely that physical activity varies for most people in the short term (e.g., on a daily basis), and the measurement of physical activity using threshold activity levels requires a longer reference time period in order to stabilize short-term fluctuations in behavior.

Construct validity hypotheses were generally supported, with correlation patterns as expected, but with correlations no more than moderate in strength. The most interesting findings emerged from the correlations between SMP-T2D measures and the construct validation measures. Validity measures were more strongly correlated with ease of performing self-management behaviors than with frequency of performing the same behaviors. This suggests that ease/difficulty is a more sensitive measure of self-management or that patients are more willing to admit to having difficulty with self-management than to admit not implementing their prescribed regimen. The one exception was for coping, but this may be because coping behavior was measured as the consequences of coping rather than the frequency of coping behavior.

The strongest findings regarding validity were obtained from the longitudinal analyses. Change in SMP-T2D measures was moderately associated with change in the validity measures, as predicted. In particular, multiple SMP-T2D measures predicted change in A1c and perceived frequency of hypoglycemia. Perhaps the most interesting finding was that two SMP-T2D measures were related to change in hypoglycemia in opposite directions; increased physical activity increased hypoglycemia and increased glucose monitoring lowered hypoglycemia.

The importance rating items did not yield any clear evidence of any one domain being more important than another; rather, all domains were deemed very to extremely important. Results may also reflect the selection of respondents into a clinical trial, as participants are likely to be those with a strong belief in the importance of treatment. Importance might be considered for further testing and inclusion in observational or non-clinical trials, as variations in patient beliefs about the importance of performing various regimen behaviors may be useful in informing clinical practice. Also, the measurement approach might be modified so that respondents *rank order* the importance of behaviors rather than *rate* them in order to increase discrimination.

### Study strengths and limitations

One of the strengths of the research reported here is that it involved multiple independent studies. Following FDA guidance, there was an initial study to obtain patient input about the measures to be included to represent the key domains, and cognitive debriefing provided patient input about item content/wording. Then an initial validation study generated information about psychometrics and was used to select final items for inclusion in the version used to assess sensitivity to change and predictive validity.

Limitations of the research reported here include the fact that participants in clinical trials tend to differ from the larger patient population in a number of ways. Moreover, neither of the validation studies was designed specifically for validation of the SMP-T2D (e.g., parallel measures of the SMP-T2D constructs were not used, reducing the size of validation correlations). A trial that attempted to change behavior in specific self-management domains (e.g., comparing usual care with an intervention designed to improve healthy eating or physical activity) might have provided a more rigorous test of the ability of the SMP-T2D to detect changes in behavior. Alternatively, a longitudinal observational study might have provided a better opportunity for testing the association between changes in SMP-T2D assessments of medication taking behavior and clinical outcomes.

In future research, it may be prudent to expand the topics covered by the SMP-T2D. Specifically, the current version assesses only one benefit of treatment – weight management. Research has shown that other aspects of treatment are important to patients, including burden of treatment (convenience, comfort, embarrassment), clinical efficacy (ability to control hyperglycemia), and safety (avoidance of hypoglycemia) [34,35]. Incorporating these dimensions into the SMP-T2D would broaden its applicability and could enhance its potential value as a research and clinical tool.

## Conclusions and implications

The psychometric performance of the SMP-T2D in these trial-based analyses suggests that it could be an important addition to the compendium of instruments used to assess diabetes self-management and its contribution to treatment outcomes. It was quickly completed by patients reading at a seventh grade level and its inclusion in the clinical trials had minimal impact on trial management.

The SMP-T2D was developed for use in clinical trials; although generalizability outside clinical trials has yet to be determined a potential strength of the instrument is that the SMP-T2D concepts are also relevant in observational research. The SMP-T2D also might have potential as a discussion tool to improve communication between patients and clinicians in choosing and implementing treatment regimens.

## Appendix 1

*Content of the Self-Management Profile for Type 2 Diabetes* (SMP-T2D)

1. How many days during the past week (last 7 days) did you miss taking your diabetes medications as prescribed? {0–7, reverse scored}

2. How many days during the past week (last 7 days) did you miss monitoring your blood sugar? {0–7, reverse scored}

3. How many days during the past week (last 7 days) did you eat foods not healthy for your diabetes? {0–7}

4. During the past week (last 7 days), how many days did you eat more food than you were supposed to? {0–7, reverse scored}

5. How many days during the past week (last 7 days), did you do at least some light physical activity (such as walking, light gardening)? {0–7}

6. How many days during the past week (last 7 days), did you do at least 30 minutes of moderate physical activity (such as pushing a vacuum cleaner, riding a bicycle, playing golf)? {0–7}

7. How many days during the past week (last 7 days), did you do at least 20 minutes of vigorous physical activity (such as running or participating in strenuous sports)? {0–7}

8. During the past week, how much difficulty did you have with: {A great deal, A lot, Moderate, A little, No}

a. monitoring your blood sugar?

b. giving yourself your diabetes medications as your doctor instructed?

c. managing your weight?

d. periods of uncontrolled eating?

e. feeling hungry?

f. food cravings?

g. being physically active?

h. coping with frustration and worry related to your diabetes?

9. During the past week (last 7 days), how frustrated have you been with trying to manage your diabetes? {Not at all, Slightly, Moderately, Very, Extremely}

10. During the past week (last 7 days), how worried have you been about your future health because of your diabetes? {Not at all, Slightly, Moderately, Very, Extremely}

11. Overall, how confident have you felt during the past week (last 7 days) about being able to manage your diabetes? {Not at all, Slightly, Moderately, Very, Extremely}

12. How important is it for you right now to: {Lot, Moderate, Little, No}

a. monitor your blood sugar?

b. take your diabetes medications as your doctor instructed?

c. manage your weight?

d. manage your diet?

e. manage your physical activity?

f. manage frustration and worry related to your diabetes?

NOTE: Question 12 was not included in the version of the SMP-T2D used in Study 2.

## Competing interests

This work was supported by Amylin Pharmaceuticals, Inc., San Diego, CA. DMB, MLM, MP and DLP have received funding from Amylin Pharmaceuticals, Inc. JHB and AC are employees of Amylin Pharmaceuticals, Inc. DLP has received honoraria from Amylin Pharmaceuticals, Inc. for participating in the development of the instrument, analysis of the data and preparation of this manuscript. MP has received honoraria from Amylin Pharmaceuticals, Inc. for participating in the analysis of the data and preparation of this manuscript.

## Authors’ contributions

MP provided input on the SMP-T2D development, participated in planning the analyses, performed statistical analyses and helped draft the manuscript. DMB performed statistical analyses and helped draft the manuscript. JHB participated in planning the study designs, participated in the SMP-T2D development, and helped draft the manuscript. MLM participated in the SMP-T2D development and helped draft the manuscript. AC helped draft the manuscript. DLP participated in the SMP-T2D development, participated in planning the analyses and helped draft the manuscript. All authors read and approved the final manuscript.
